# Computational Screening of MOFs for Acetylene Separation

**DOI:** 10.3389/fchem.2018.00036

**Published:** 2018-02-27

**Authors:** Ayda Nemati Vesali Azar, Seda Keskin

**Affiliations:** Department of Chemical and Biological Engineering, Koc University, Istanbul, Turkey

**Keywords:** metal organic frameworks, C_2_H_2_ separation, adsorption, selectivity, molecular simulation

## Abstract

Efficient separation of acetylene (C_2_H_2_) from CO_2_ and CH_4_ is important to meet the requirement of high-purity acetylene in various industrial applications. Metal organic frameworks (MOFs) are great candidates for adsorption-based C_2_H_2_/CO_2_ and C_2_H_2_/CH_4_ separations due to their unique properties such as wide range of pore sizes and tunable chemistries. Experimental studies on the limited number of MOFs revealed that MOFs offer remarkable C_2_H_2_/CO_2_ and C_2_H_2_/CH_4_ selectivities based on single-component adsorption data. We performed the first large-scale molecular simulation study to investigate separation performances of 174 different MOF structures for C_2_H_2_/CO_2_ and C_2_H_2_/CH_4_ mixtures. Using the results of molecular simulations, several adsorbent performance evaluation metrics, such as selectivity, working capacity, adsorbent performance score, sorbent selection parameter, and regenerability were computed for each MOF. Based on these metrics, the best adsorbent candidates were identified for both separations. Results showed that the top three most promising MOF adsorbents exhibit C_2_H_2_/CO_2_ selectivities of 49, 47, 24 and C_2_H_2_/CH_4_ selectivities of 824, 684, 638 at 1 bar, 298 K and these are the highest C_2_H_2_ selectivities reported to date in the literature. Structure-performance analysis revealed that the best MOF adsorbents have pore sizes between 4 and 11 Å, surface areas in the range of 600–1,200 m^2^/g and porosities between 0.4 and 0.6 for selective separation of C_2_H_2_ from CO_2_ and CH_4_. These results will guide the future studies for the design of new MOFs with high C_2_H_2_ separation potentials.

## Introduction

Metal organic frameworks (MOFs), nanoporous materials that are composed of metal clusters connected with organic linkers, have attracted significant interest in the last decade. MOFs offer a wide range of pore sizes, permanent porosities, very large surface areas, and good chemical stabilities (Li et al., 1999; Eddaoudi et al., 2002). The most important characteristic of MOFs is that their physical, chemical and structural properties can be tuned during synthesis. This controllable synthesis leads to a large diversity of materials having different geometry, pore size, and chemical functionality (Mondloch et al., 2013). Due to these advantageous physical and chemical properties, MOFs have emerged as strong alternatives to traditional nanoporous materials in various gas separation applications. MOFs have been widely examined for CO_2_ separation because of the growing environmental concerns on the removal of CO_2_ from natural gas (CO_2_/CH_4_), flue gas (CO_2_/N_2_), and from other gases (CO_2_/H_2_). Experimentally measured selectivities and gas uptake capacities of several MOFs for separation of CO_2_ from CH_4_ and N_2_ have been reported and results showed that MOFs can be strong alternatives to traditional porous materials in CO_2_ separations (Li et al., 2011). Comparison of CO_2_ separation performances of MOFs, zeolites and activated carbons showed that CO_2_/N_2_ selectivity changes from low in zeolites to moderate in carbon-based absorbents and becomes high in MOFs (Ben-Mansour et al., 2016). Although a significant number of studies exist on the CO_2_ separation with MOFs, acetylene separation with these new porous materials has not been thoroughly investigated and research on MOFs for acetylene separation left behind that for CO_2_ separation.

Acetylene (C_2_H_2_) is the simplest member of unsaturated hydrocarbons and it is produced by different processes such as reaction of water with calcium carbide from coal, partial oxidation of natural gas, or as a byproduct of ethylene steam cracking (Zhang et al., 2011). C_2_H_2_ is a very important raw material for the synthesis of various industrial chemicals such as polyurethane and polyester plastics, consumer products, and oxy-acetylene welding and cutting in metal fabrication. Since high purity C_2_H_2_ is strongly needed for these processes, C_2_H_2_ separation is important in the industry. C_2_H_2_ is traditionally separated from CO_2_ and CH_4_ using cryogenic distillation, however this process is very costly. C_2_H_2_/CO_2_ separation is specifically challenging because both gas molecules have similar molecular sizes (3.4 × 3.4 × 5.5 and 3.4 × 3.4 × 5.3 Å) and boiling points (189.3 and 194.7 K) (Foo et al., 2016). The energy and equipment costs associated with these gas separations could be significantly reduced by the development of alternative separation methods such as adsorption-based gas separations which provide very large reductions in energy consumption and costs of these processes. The greatest limitation in the applications of adsorption-based gas separation technologies is the low selectivity of the materials used as adsorbents. Therefore, identification of new adsorbent materials that can achieve C_2_H_2_ separation from other gases with high selectivity has gained significant attention.

An ideal adsorbent material should offer a good combination of high adsorption selectivity and high uptake capacity in addition to good stability. There is a wide range for C_2_H_2_ uptake capacities of MOFs from 25 to 200 cm^3^/g reported at 1 bar and 298 K (Zhang et al., 2011). Recent studies on MOFs showed that it is difficult to simultaneously achieve both high C_2_H_2_/CO_2_ selectivity and high C_2_H_2_ uptake capacity (Wen et al., 2016). For example, a widely studied MOF, HKUST-1 (also known as CuBTC in the MOF literature) was reported to exhibit high C_2_H_2_ uptake, 201 cm^3^/g at 1 bar and 298 K (Xiang et al., 2009). However, its C_2_H_2_/CO_2_ selectivity was found to be low, 6, based on the ideal adsorbed solution theory (IAST) calculations (Myers, 2002) for equimolar C_2_H_2_/CO_2_ mixture (Li et al., 2014). A MOF named as UTSA-50 was shown to exhibit higher C_2_H_2_/CO_2_ selectivity, 13.3 at 1 bar and 296 K based on the Henry's law ratios of C_2_H_2_ and CO_2_ but its C_2_H_2_ uptake was low, 91 cm^3^/g, which was attributed to its low surface area (Xu et al., 2013). Wen et al. (2016) synthesized a new MOF and measured its C_2_H_2_ uptake as 216 cm^3^/g at 1 bar and 298 K, which was one of the highest C_2_H_2_ uptakes of MOFs reported to date. They also calculated the C_2_H_2_/CO_2_ selectivity of the MOF using IAST based on the single-component adsorption isotherms data of C_2_H_2_ and CO_2_. Results showed that C_2_H_2_ selectivity is 11.5 at 1 bar for separation of an equimolar C_2_H_2_/CO_2_ mixture. Li et al. (2014) studied C_2_H_2_/CO_2_ separation performance of hydrogen bonded organic frameworks (HOFs) and calculated selectivity of HOF-3 using IAST as 21 at 1 bar and 296 K. Isostructural MOF-74 materials having different metal sites were reported to have high C_2_H_2_ uptakes, 120–197 cm^3^/g at 1 bar and 295 K, however their C_2_H_2_/CO_2_ selectivities were not reported. Xiang et al. (2010) studied the effect of metal sites on C_2_H_2_ storage performance of four isostructural MOFs and reported the highest C_2_H_2_ uptake capacity as 198 cm^3^/g. Similar to C_2_H_2_/CO_2_ separation, C_2_H_2_/CH_4_ separation is an important process because C_2_H_2_ is mainly derived from the cracking of crude oil and residual oils. Purification is necessary to meet the requirement of high-purity C_2_H_2_ for the organic synthesis (Zhang et al., 2011). Separation of C_2_H_2_ from CH_4_ using MOFs has been very rarely studied in the literature. UTSA-50 was reported to have a high C_2_H_2_/CH_4_ selectivity, 68, based on the ratio of Henry's constants of gases (Xu et al., 2013).

As can be seen from this literature review, most experiments only reported the C_2_H_2_ uptake of MOFs and estimated MOFs' selectivities using the single-component data without performing the adsorption measurements for gas mixtures. In reality, gases exist as mixtures and selectivities should be calculated for gas mixtures. Considering the large number and variety of available MOFs, it is very challenging to identify the most promising MOF materials for adsorption-based separation of C_2_H_2_/CO_2_ and C_2_H_2_/CH_4_ mixtures using purely experimental manners. Molecular simulations play an important role in studying adsorption of various gas molecules in a large number of MOFs in a time effective manner to identify the best materials for a target gas separation (Colón and Snurr, 2014). There are some computational studies on C_2_H_2_ storage performance of MOFs in the literature (Pang et al., 2015; Chen et al., 2016; Zhang et al., 2017) however, the number of molecular simulation studies on C_2_H_2_ separation is very limited. Fischer et al. (2010) performed the first molecular simulation study to obtain adsorption isotherm of equimolar C_2_H_2_/CO_2_ mixture in HKUST-1. The C_2_H_2_/CO_2_ selectivity was calculated from the mixture data as 2.4, which was much lower than the one calculated from the single-component adsorption data (6). Yeganegi et al. (2017) carried out Grand Canonical Monte Carlo (GCMC) simulations for adsorption of equimolar C_2_H_2_/CH_4_ mixture in MOF-5, MOF-505, and HKUST-1. The C_2_H_2_/CH_4_ selectivity of HKUST-1 (66) was computed to be significantly higher than that of MOF-505 (6) and MOF-5 (2) at 1 bar and 295 K. Ji et al. (2017) recently performed GCMC simulations to calculate adsorption isotherms for single-component C_2_H_2_, CO_2_, CH_4_, and equimolar C_2_H_2_/CO_2_ and C_2_H_2_/CH_4_ mixtures at 298 K. They considered 11 MOFs having the same metal and showed that all MOFs except MOF-505 have C_2_H_2_/CH_4_ selectivities lower than 7 and C_2_H_2_/CO_2_ selectivities lower than 2. Selectivity of MOF-505 for C_2_H_2_/CH_4_ was computed to be around 9 whereas selectivity for C_2_H_2_/CO_2_ was found to be <2 at 1 bar. As can be seen from this review, current molecular simulation studies calculated C_2_H_2_ selectivities of at most 11 different MOF structures having the same metal. There is no large-scale computational screening study to assess C_2_H_2_/CO_2_ and C_2_H_2_/CH_4_ selectivities of MOFs that span a large variety in structural properties. It is also important to note that although selectivity is a widely used metric to assess the gas separation performances of adsorbents, several other metrics such as working capacity and regenerability determine the practical usability of MOF adsorbents in separation processes. These metrics have not been examined for adsorption-based C_2_H_2_ separation performances of MOFs to date.

In this work, we performed molecular simulations for a large number and variety of MOFs to examine their separation potentials for C_2_H_2_/CO_2_ and C_2_H_2_/CH_4_ mixtures. Adsorption data of C_2_H_2_/CO_2_ and C_2_H_2_/CH_4_ mixtures obtained from the GCMC simulations were used to calculate several adsorbent performance metrics of MOFs including adsorption selectivity, working capacity, adsorbent performance score (APS), sorbent selection parameter and regenerability. Separation performances of MOFs were evaluated based on these metrics and the top performing MOF adsorbents were identified for C_2_H_2_/CO_2_ and C_2_H_2_/CH_4_ separations. We then examined the relations between structural properties of MOFs such as pore sizes, porosities, surface areas and their C_2_H_2_ selectivities to provide the structure-performance relationships that can serve as a map for experimental synthesis of new MOFs with better C_2_H_2_ separation performances.

## Materials and methods

### MOFs

We used the MOFs that represent a large variety in structure and chemistry from our previous work (Sumer and Keskin, 2016) and the crystallographic information of these MOFs were obtained from the literature (Chung et al., 2014). We also included some newly synthesized MOFs for which experimental C_2_H_2_ uptakes were reported and crystallographic information of these MOFs were taken from the Cambridge Structural Database (CSD) (Groom and Allen, 2014). As a result, we considered 174 different MOF structures in this work. Structural properties of MOFs such as pore limiting diameter (PLD), the largest cavity diameter (LCD), accessible surface area, pore volume and density were calculated using Zeo++ software (Willems et al., 2012). We only considered MOFs with LCDs larger than 4 Å so that all three gas molecules can be adsorbed in the pores of materials. The PLDs, LCDs, surface areas, pore volumes, and densities of MOFs range from 2.5 to 15.6 Å, 4.1 to 28.7 Å, 103 to 5,800 m^2^/g, 0.08 to 3.3 cm^3^/g, and 0.18 to 5.05 g/cm^3^, respectively. The complete list of the MOFs studied in this work and their calculated structural properties are given in Table S1.

### Simulation details

We performed GCMC simulations to compute adsorption isotherms of gas mixtures in MOFs (Frenkel and Smit, 2002). These simulations were carried out as implemented in the RASPA simulation code (Dubbeldam, 2014). Five different types of moves, translation, reinsertion, rotation, swap, and identity exchange of molecules were considered. The Lorentz-Berthelot mixing rules were employed. The cut-off distance for truncation of the intermolecular interactions was set to 12.5 Å. The simulation cell lengths were increased to at least 25 Å along each dimension and periodic boundary conditions were applied in all simulations. For each MOF, simulations were carried out for 60,000 cycles with the first 10,000 cycles for initialization. Figure S1 shows that molecular simulation reached equilibrium at 10,000 cycles and increasing the cycle number does not affect the number of adsorbed gas molecules. Peng-Robinson equation of state was used to convert the pressure to the corresponding fugacity. More details of these simulations can be found in the literature (Frenkel and Smit, 2002; Dubbeldam, 2014).

C_2_H_2_ molecule was represented as a two-site rigid and linear model with the Lennard-Jones (LJ) positions located on the carbon atoms and partial charges located on each atom (Fischer et al., 2010). Unsaturated C=C and C–H bond lengths were considered as 1.211 and 1.071 Å, respectively. CO_2_ molecule was modeled as a linear molecule with three LJ sites and partial charges were centered on each atom (Potoff and Siepmann, 2001). The rigid C–O bond length used in this model was 1.16 Å. Single-site spherical LJ 12–6 potential was used to model CH_4_ molecules (Chen and Siepmann, 1999). All LJ parameters and atomic partial charges of gas molecules are given in Table S2.

The potential parameters of MOF atoms were taken from the Universal Force Field (UFF) (Rappé et al., 1992) since UFF has been successful in predicting gas adsorption and separation performances of a large number of MOFs in previous studies (Keskin et al., 2009). Furthermore, UFF contains potential parameters for all elements of the periodic table and applicable to all types of MOFs having a variety of atoms. Potential parameters of Cu atoms of MOFs were taken from a molecular simulation study (Fischer et al., 2010) in which modified parameters for Cu was shown to better represent the interaction of carbon site of C_2_H_2_ and oxygen site of CO_2_ with the unsaturated Cu sites of MOFs. Electrostatic interactions were taken into account using the Coulomb potential. In order to compute the electrostatic interactions between gas molecules and MOFs, partial point charges were assigned to MOF atoms using the extended charge equilibration method (EQeq; Wilmer et al., 2012). MOFs were assumed to be rigid in their reported crystallographic structures in the simulations. This assumption has been used in all large-scale molecular simulation studies of MOFs to save significant computational time. Since we only considered the MOFs that have pore sizes larger than the kinetic diameters of the gas molecules, flexibility is expected to have a negligible effect on the gas adsorption results. All GCMC simulations were performed at an adsorption pressure of 1 bar and desorption pressure of 0.1 bar at 298 K since the compression limit for the safe storage of C_2_H_2_ is 2 bar. These conditions were chosen to mimic vacuum swing adsorption process following the literature (Bae and Snurr, 2011). Equimolar C_2_H_2_/CO_2_ and C_2_H_2_/CH_4_ mixtures were considered in all molecular simulations. It was recently discussed that the two most important factors in molecular simulations are the force field and the degree of sampling in the relevant configuration space (van Gunsteren et al., 2017). In order to show the good sampling of our simulations, we reported the deviations of GCMC results for the top three promising MOFs for C_2_H_2_/CO_2_ and C_2_H_2_/CH_4_ separations in Table S3 and results showed that the uncertainty for the simulated C_2_H_2_ adsorption is <3%.

### Adsorbent evaluation metrics

Results obtained from GCMC simulations were used to compute several adsorbent evaluation metrics that are defined in Table 1. Adsorption selectivity (S_ads_) is the most widely used metric to evaluate adsorbents and it is simply defined as the ratio of compositions of the adsorbed gases (x) in the adsorbent normalized by the ratio of bulk phase compositions (y). The subscript 1 represents the strongly adsorbed gas and subscript 2 represents the weakly adsorbed gas. Since the aim of our work is to identify the MOFs that are able to selectively separate C_2_H_2_ from CO_2_ and CH_4_, we reported C_2_H_2_/CO_2_ and C_2_H_2_/CH_4_ selectivities of MOFs. Working capacity (ΔN) is defined as the difference between the gas uptakes (N) at the adsorption and desorption pressures in the unit of mol gas per kg adsorbent (Bae and Snurr, 2011). We computed C_2_H_2_ working capacity of all MOFs. APS was defined as the product of selectivity and working capacity to easily identify the top performing adsorbent materials that combine high selectivities with high working capacities (Chung et al., 2016). Sorbent selection parameter (S_sp_) includes the ratio of working capacities and selectivities computed at adsorption and desorption pressures and it is useful for studying the performance of adsorbents in pressure swing adsorption processes (PSA; Rege and Yang, 2001). Per cent regenerability (R%) describes the regeneration of the adsorption sites and shows the reusability of the adsorbent in the cyclic processes (Bae and Snurr, 2011).

**Table 1:** Descriptions of adsorbent evaluation metrics.

**Metric**	**Equation**
Selectivity	${\text{S}}_{\text{ads}\left(1/2\right)}=\frac{{\text{x}}_{1}/{\text{x}}_{2}}{{\text{y}}_{1}/{\text{y}}_{2}}$
Working capacity	ΔN = N_ads_ − N_des_
Adsorbent performance score	APS = S_ads_ × ΔN
Sorbent selection parameter	${\text{S}}_{\text{sp}}=\frac{{{\text{S}}_{\text{ads},1}}^{2}}{{\text{S}}_{\text{des},1}}\times \frac{\Delta {\text{N}}_{1}}{\Delta {\text{N}}_{2}}$
Regenerability	$\text{R}\left(\%\right)=\frac{\Delta \text{N}}{{\text{N}}_{\text{ads}}}\times 100\%$

## Results and discussions

**Figure 1:**
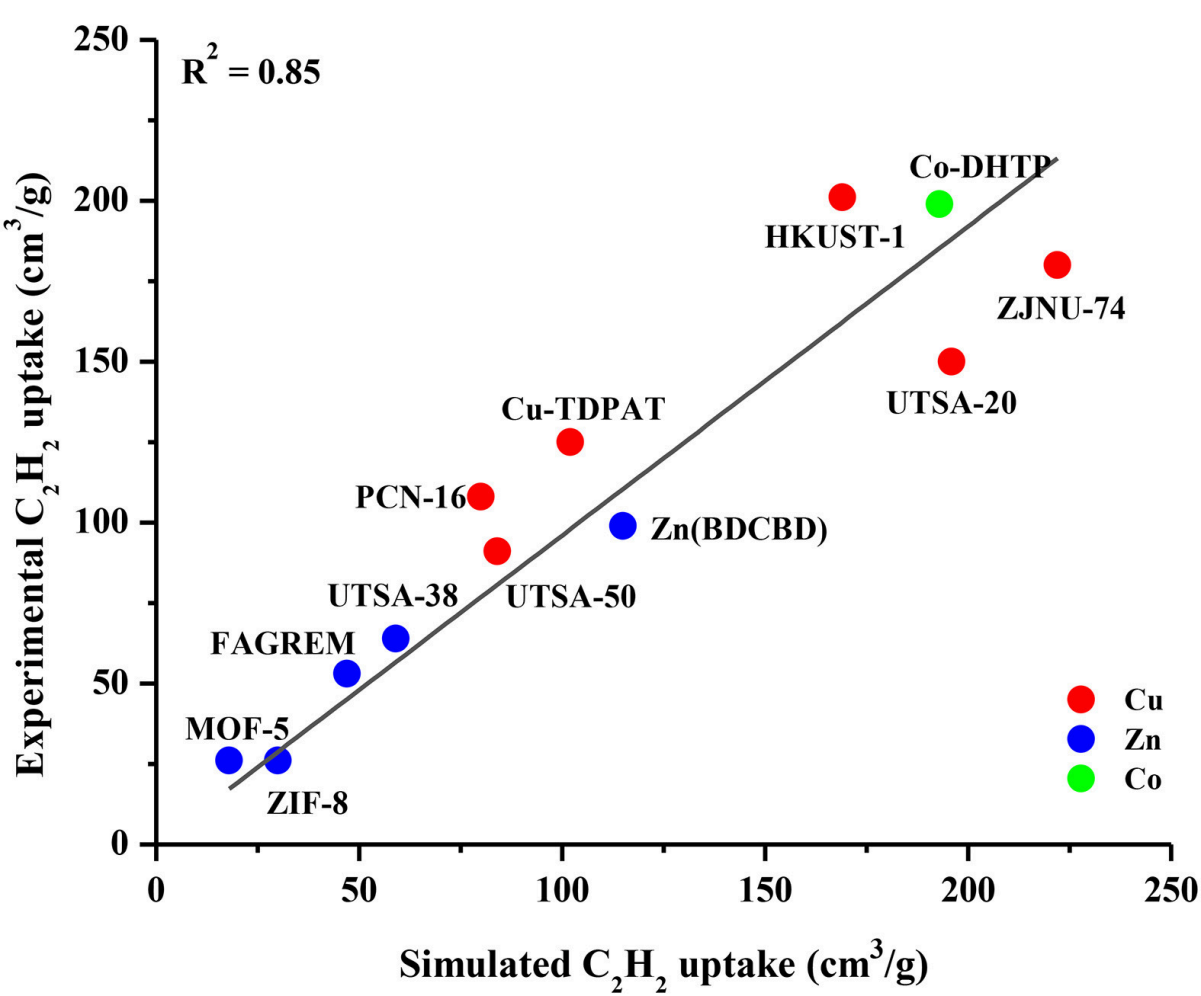
Comparison of experimental and simulated C_2_H_2_ uptakes of MOFs at 1 bar, 298 K.

The accuracy of our GCMC simulations to assess the adsorption of CO_2_ and CH_4_ molecules in various MOFs was validated in our previous works (Sezginel et al., 2015; Altintas and Keskin, 2016; Sumer and Keskin, 2016) by comparing the results of our molecular simulations with the available experimental data from different research groups. In this work, we aim to validate the GCMC simulations for C_2_H_2_ adsorption in MOFs. We collected experimental C_2_H_2_ uptake data of several different MOFs from the literature and performed GCMC simulations for these MOFs under the same conditions with the experiments at 1 bar and 298 K (in some cases 296 K). We specifically included the widely studied MOFs such as MOF-5 (IRMOF-1), HKUST-1, ZIF-8, UTSA-50 and the MOFs identified as promising due to their high C_2_H_2_ uptakes such as Co-DHTP in this comparison. Figure 1 shows that there is a good agreement between single-component adsorption experiments and simulations for C_2_H_2_ uptakes of different MOFs, indicating the appropriate choice of the force fields used in the simulations. The good agreement between experimentally reported and simulated C_2_H_2_ adsorption isotherms of IRMOF-1 and HKUST-1 up to 1 bar is also shown in Figure S2. There was no experimental data on adsorption of equimolar C_2_H_2_/CO_2_ and C_2_H_2_/CH_4_ mixtures in the literature to the best of our knowledge, therefore it was not possible to make a comparison for the mixture adsorption. The good agreement we showed in Figure 1 suggests that molecular simulations can be used to make accurate estimates about the adsorption of C_2_H_2_/CO_2_ and C_2_H_2_/CH_4_ mixtures in MOFs.

**Figure 2:**
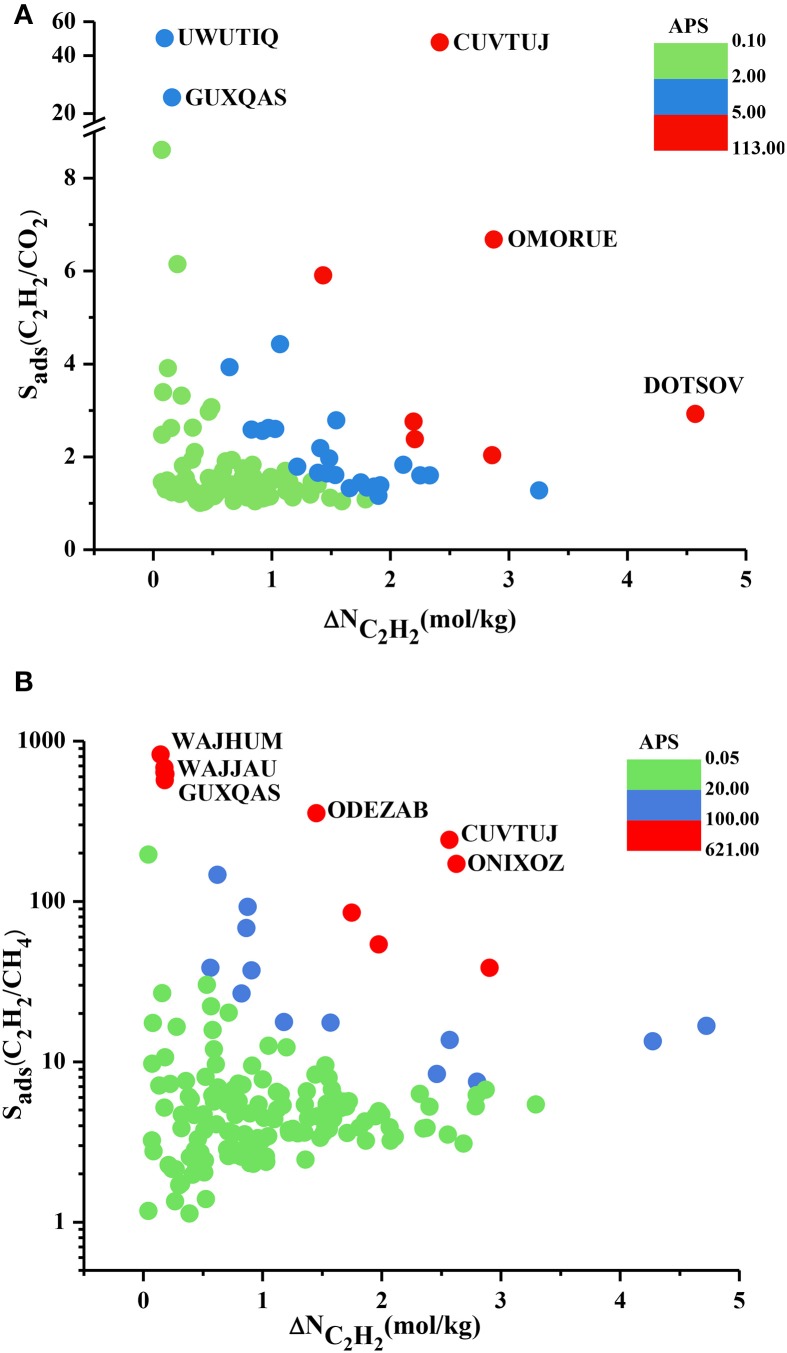
C_2_H_2_ selectivity and C_2_H_2_ working capacity of MOFs for separation of equimolar **(A)** C_2_H_2_/CO_2_ **(B)** C_2_H_2_/CH_4_ mixtures.

We performed the GCMC simulations of MOFs at 0.1 and 1 bar considering equimolar C_2_H_2_/CO_2_ and C_2_H_2_/CH_4_ mixtures and computed their C_2_H_2_ selectivities and working capacities. All 174 MOFs were found to be C_2_H_2_ selective for C_2_H_2_/CH_4_ separation whereas 121 MOFs were found to be C_2_H_2_ selective for C_2_H_2_/CO_2_ separation. Since our aim is to identify C_2_H_2_ selective MOFs in this work, we only show these 121 MOFs in Figure 2A. Figure 2A shows that most of the MOFs have C_2_H_2_/CO_2_ selectivities lower than 2. These low selectivities can be attributed to the similarity of the C_2_H_2_ and CO_2_ molecules which makes the adsorption-based C_2_H_2_/CO_2_ separation challenging. There are 25 MOFs that show C_2_H_2_/CO_2_ selectivities in the range of 2–10 and widely studied HKUST-1 is among these MOFs. We calculated its selectivity as 2.9 which is in good agreement with the literature value of 2.4 (Fischer et al., 2010). This slight difference can be attributed to (a) the different potential parameters used for CO_2_, (b) the different charge assignment methods used for CuBTC and/or (c) different crystal structures of CuBTC used in two simulation studies. Three MOFs, UWUTIQ, CUVTUJ [also known as Co_2_(DHTP)] and GUXQAS, were found to show the highest C_2_H_2_/CO_2_ selectivities of 49, 47, and 24, respectively. The highest C_2_H_2_/CO_2_ selectivities were reported for UTSA-50 and HOF-3 using IAST calculations as 13 and 21, respectively (Xu et al., 2013; Li et al., 2014). The three MOFs mentioned above outperform UTSA-50 and HOF-3 in terms of selectivity. On the other hand, Figure 2A shows that MOFs with high C_2_H_2_/CO_2_ selectivities generally suffer from low C_2_H_2_ working capacities. A large number of MOFs was found to exhibit C_2_H_2_ working capacities lower than 2 mol/kg. The best MOF adsorbents are expected to offer both high C_2_H_2_ selectivities and high C_2_H_2_ working capacities. In order to identify the MOFs that offer a good combination of C_2_H_2_ selectivity and C_2_H_2_ working capacity, we color-coded Figure 2A with APS values to separate low and high-performance regions within the MOF search space. Three different regions were defined to provide a reference for quantitatively identifying a number of promising MOFs. MOFs with high C_2_H_2_ selectivity but low C_2_H_2_ working capacity and MOFs with low C_2_H_2_ selectivity but high C_2_H_2_ working capacity were located in the green region. These MOFs have APS values lower than 2 for separation of equimolar C_2_H_2_/CO_2_ mixtures. MOFs showing moderate selectivities (1.2–49) and working capacities (0.1–3.25 mol/kg) result in APS values of 2.14–4.76 and they are shown in the blue region. Finally, the most promising MOFs with the best selectivity and working capacity combinations are shown in the red region with APS > 5.25. There are 7 MOFs in this region and except one all have C_2_H_2_/CO_2_ selectivities in the range of 2–7 and their working capacities are between 1.5 and 3 mol/kg. Three MOFs with the highest APSs, CUVTUJ, OMORUE, and DOTSOV can be considered as the best candidates for C_2_H_2_/CO_2_ separations.

Figure 2B shows that C_2_H_2_/CH_4_ selectivities of MOFs have a wide range from 1.2 to 824, and most of the MOF adsorbents have C_2_H_2_ selectivities lower than 10. The C_2_H_2_ working capacities of MOFs were also calculated to span a wide range, from 0.04 to 4.7 mol/kg. For equimolar C_2_H_2_/CH_4_ mixture, Alduhaish et al. (2017) reported the highest selectivity, 98, for a MOF (VAQXUJ) using IAST at 1 bar and 296 K. Ten MOFs we studied exhibit higher selectivities than this record. The top three selective MOFs were identified to be WAJHUM, WAJJAU, GUXQAS with C_2_H_2_/CH_4_ selectivities of 824, 684, 638, respectively. These MOFs outperform the widely studied MOFs such as HKUST-1 and UTSA-50 which were reported to have C_2_H_2_/CH_4_ selectivities of ~66–68 at 1 bar, 295/296 K as we discussed above. The four MOFs with the highest selectivities suffer from low C_2_H_2_ working capacities, ~0.17 mol/kg. The combination of high selectivity and low working capacity of these MOFs can be attributed to their relatively low pore volumes (<0.45 cm^3^/g). CUVTUJ and ONIXOZ exhibit both high C_2_H_2_/CH_4_ selectivities (242 and 171, respectively) and high C_2_H_2_ working capacities (~2.6 mol/kg). Similar to Figure 2A, we color-coded the APSs of MOFs where green represents the MOFs with low performance (APSs < 20), blue represents the promising MOFs with C_2_H_2_/CH_4_ selectivities of 7.5–147, C_2_H_2_ working capacities of 0.6–5 mol/kg, resulting in 20 < APSs < 92. Finally, the most promising MOFs for separation of equimolar C_2_H_2_/CH_4_ mixtures are located in the red region with APSs > 102. The best candidates for C_2_H_2_/CH_4_ separations were identified as CUVTUJ, ONIXOZ (high selectivity and high working capacity), and ODEZAB (high selectivity and moderate working capacity). These MOFs were computed to have APSs of 621, 450, and 515, respectively.

At that point it is also useful to compare the C_2_H_2_ working capacities of MOFs with each other. Zhang et al. (2017) calculated single-component C_2_H_2_ working capacities of 7 MOF structures as 110–180 cm^3^ (STP)/g at an adsorption pressure of 1 bar and desorption pressure of 0.1 bar. The highest C_2_H_2_ working capacities of the 174 MOFs we considered in this work were calculated to be as 103 cm^3^ (STP)/g and 106 cm^3^ (STP)/g for C_2_H_2_/CO_2_ and C_2_H_2_/CH_4_, respectively, under the same conditions. These values belong to a well-known MOF, HKUST-1. It is important to note that our C_2_H_2_ working capacities were calculated for equimolar mixtures, not for the single-component gas adsorption and therefore they are less than the ones reported for single-component cases.

**Figure 3:**
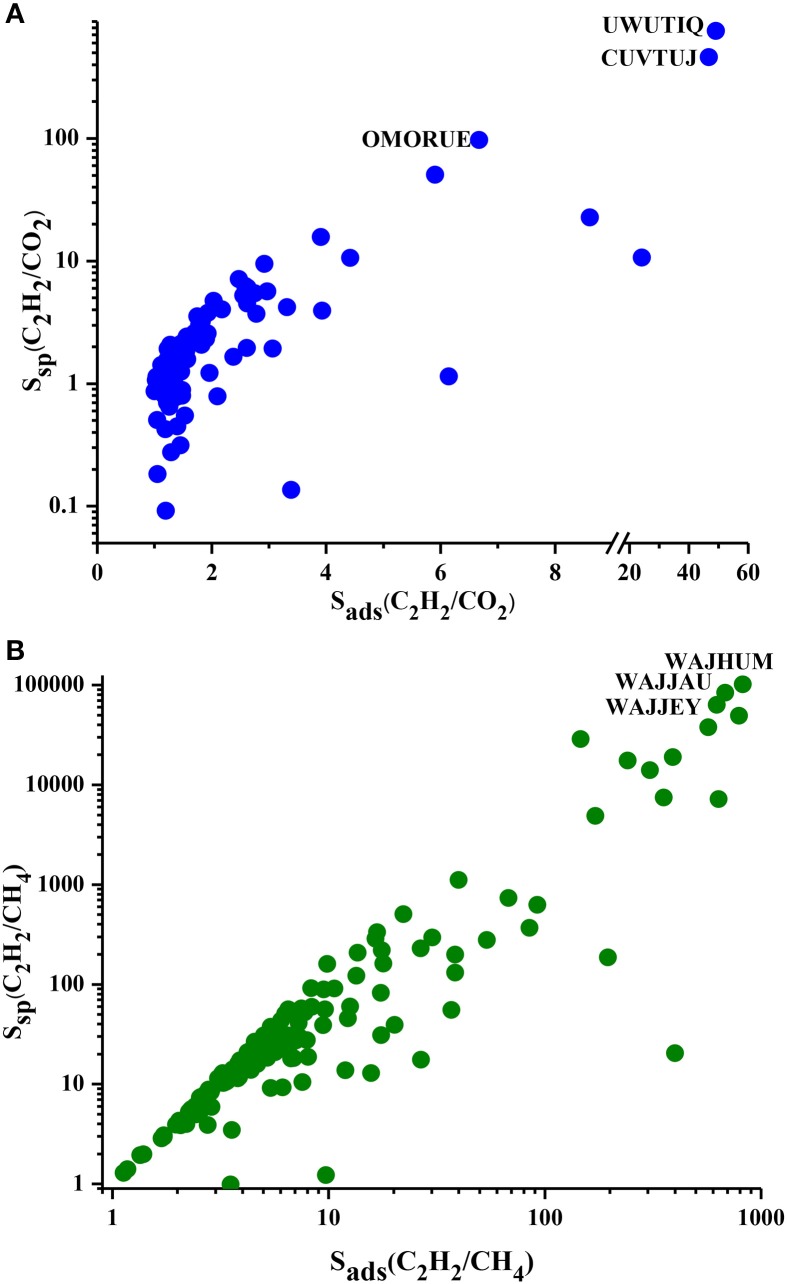
S_sp_ and S_ads_ of MOFs for **(A)** C_2_H_2_/CO_2_ and **(B)** C_2_H_2_/CH_4_ separations.

We showed the S_sp_ values of MOFs for separation of C_2_H_2_/CO_2_ and C_2_H_2_/CH_4_ mixtures in Figure 3 as a function of selectivity. S_sp_ values of MOF adsorbents are in the range of 0.1–755 and 1.2–101,362 for C_2_H_2_/CO_2_ and C_2_H_2_/CH_4_ separations, respectively. S_sp_ increases with selectivity as described in Table 1. Most of the MOFs have S_sp_ values lower than 10 for C_2_H_2_/CO_2_ separation due to the low selectivities as shown in Figure 3A. S_sp_ values of MOFs are <100 for C_2_H_2_/CH_4_ separation as shown in Figure 3B. It is important to note that a MOF which has been widely studied as adsorbent in the literature, IRMOF-1 (SAHYIK), has a low S_sp_ (3.1) for C_2_H_2_/CH_4_ separation. This means there are many other MOFs with better separation potentials than this widely studied MOF. The most promising MOFs are located at the top right corner of Figure 3 which have both high S_sp_ and high selectivity such as UWUTIQ (S_sp_: 754.88, S_ads_:49) for C_2_H_2_/CO_2_ and WAJHUM for C_2_H_2_/CH_4_ (S_sp_: 101,36 and S_ads_: 824). To summarize, APS identifies CUVTUJ, OMORUE, and DOTSOV (CUVTUJ, ODEZAB, and ONIXOZ) as the best adsorbents for C_2_H_2_/CO_2_ (C_2_H_2_/CH_4_) separation whereas S_sp_ identifies UWUTIQ, CUVTUJ, and OMORUE (WAJHUM, WAJJAU, and WAJJEY) as the best adsorbents for C_2_H_2_/CO_2_ (C_2_H_2_/CH_4_) separation. These results show that for C_2_H_2_/CO_2_, CUVTUJ, and OMORUE are the promising adsorbents based on both APS and S_sp_. The low working capacity of UWUTIQ, which has the highest selectivity and S_sp_, leads to a low APS value. MOFs that are promising for separation of C_2_H_2_/CH_4_ were identified to be different based on these two metrics because MOFs with high selectivities generally have low working capacities (as shown in Figure 2B) resulting in high S_sp_ but low APS values.

**Figure 4:**
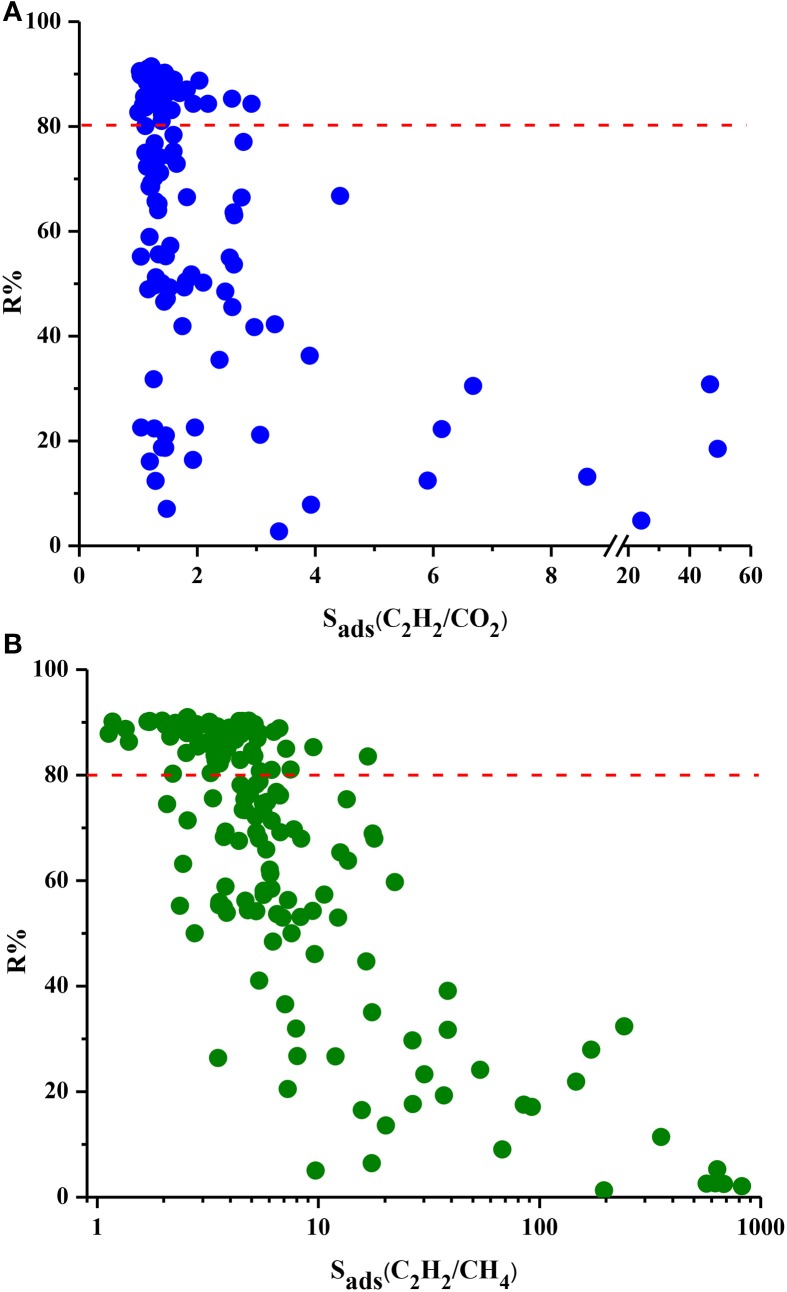
R% and S_ads_ of MOFs for **(A)** C_2_H_2_/CO_2_ and **(B)** C_2_H_2_/CH_4_ mixtures.

Figure 4 shows the relation between R% and selectivity of MOFs. MOFs show a very wide range of R%, from 2 to 91% for these two mixtures and the red dotted line represents R% = 80%. We chose this value as the minimum desired R% since lower R% values result in high cost in adsorption-based gas separation applications (Li et al., 2009). There is generally an inverse relation between R% and S_ads_. As the MOF strongly adsorbs one gas component over other, desorption becomes difficult resulting in low working capacities and low regenerabilities. Almost half of the MOFs exhibit R% higher than 80%, however these MOFs have low selectivities. For example, the three most selective MOFs for C_2_H_2_/CO_2_ separation have very low R% values of 18, 31, and 5% as can be seen from Figure 4A. Similarly, three MOFs with the highest C_2_H_2_/CH_4_ selectivities suffer from very low R% (2, 3, and 5%, respectively) as shown in Figure 4B. These results show that choosing the best adsorbent material based on solely selectivity is not completely accurate and other metrics such as R% should be considered. Among the MOFs which have R% higher than 80%, DOTSOV shows the highest selectivity of 3 and 17 for C_2_H_2_/CO_2_ and C_2_H_2_/CH_4_ mixtures, respectively. This MOF also exhibits high C_2_H_2_ working capacity for C_2_H_2_/CO_2_ and C_2_H_2_/CH_4_ (4.6 and 4.73 mol/kg) suggesting that it is a promising adsorbent for both separations considering the cost of regeneration.

**Figure 5:**
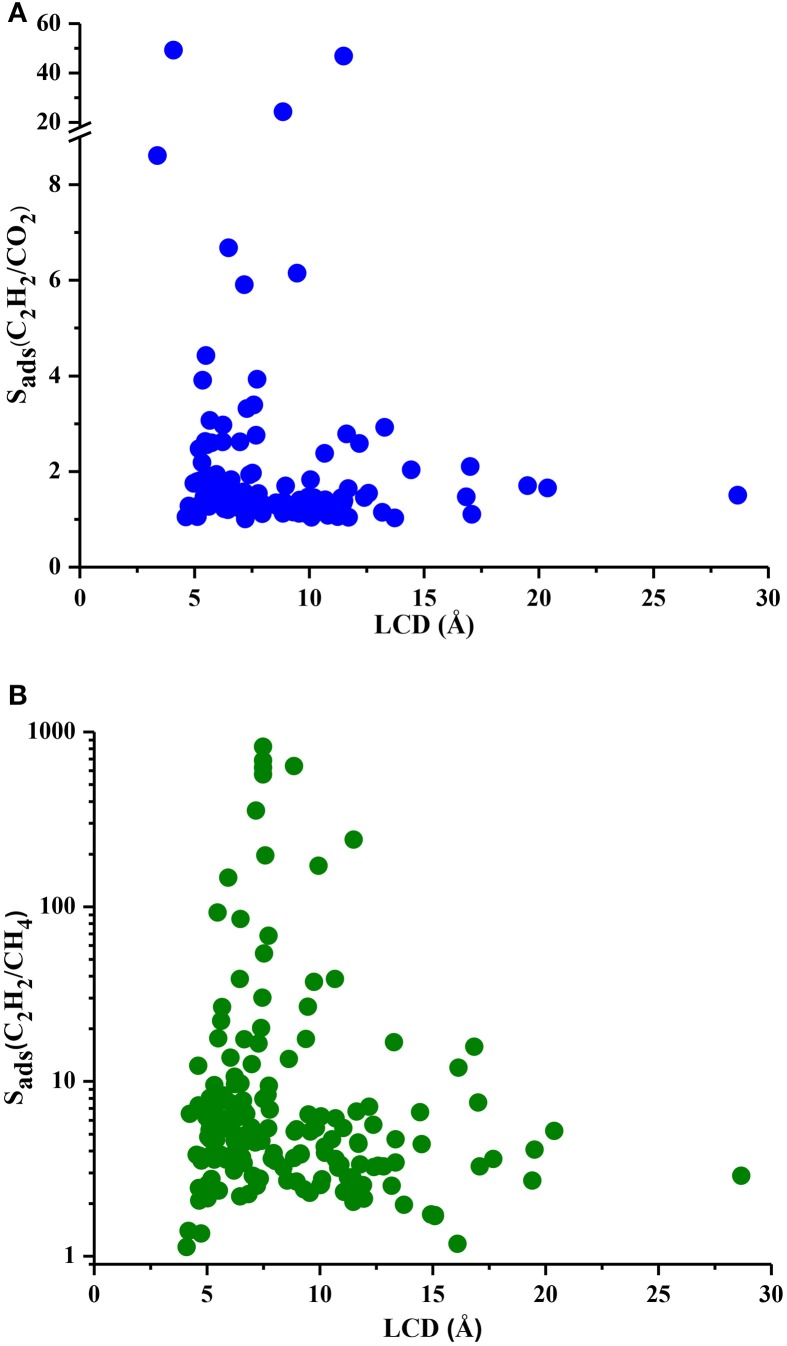
S_ads_ and LCD of MOFs for **(A)** C_2_H_2_/CO_2_ and **(B)** C_2_H_2_/CH_4_ mixtures.

**Figure 6:**
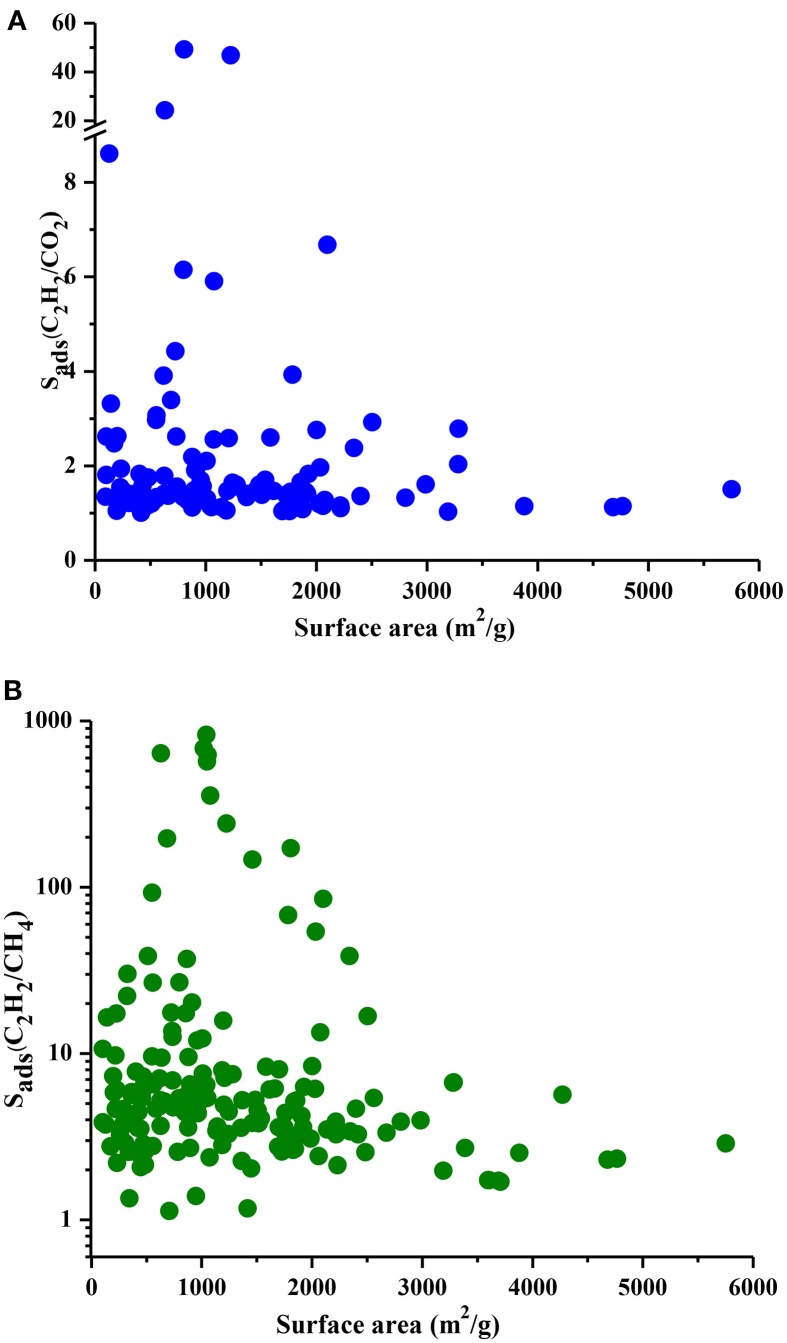
S_ads_ and surface areas of MOFs for **(A)** C_2_H_2_/CO_2_ and **(B)** C_2_H_2_/CH_4_ mixtures.

Understanding the correlations between separation performances of MOF adsorbents and their structural properties is crucial for identification of the best candidates with pre-determined structural features. Since selectivity is the most widely considered metric in choosing adsorbents, we examined the relation between LCDs of MOFs and their selectivities in Figure 5. Results show that MOFs having pores in the range of 5–10 Å generally have high selectivities both for C_2_H_2_/CO_2_ and C_2_H_2_/CH_4_ separations whereas MOFs with LCDs > 10 Å exhibit low adsorption selectivities. This result is in agreement with the findings of a recent study in which MOFs with LCDs of 6.7–10 Å were found to have the highest single-component C_2_H_2_ uptakes (Zhang et al., 2017). Figure 6 shows the relation between accessible surface areas of MOFs and their selectivities. MOFs having surface areas between 180 and 1,200 m^2^/g have higher C_2_H_2_/CO_2_ selectivities whereas MOFs with surface areas of 550–1,800 m^2^/g are more promising for selective separation of C_2_H_2_ from CH_4_. Although there is not a very strong relation between surface area and selectivity, Figure 6B suggests that MOFs with large surface areas (>3,500 m^2^/g) are not very selective. We also investigated the relation between APSs and accessible surface areas of MOFs in Figure S3. Similar to selectivity, there is not an obvious relation but MOFs having high APSs generally have surface areas of 1,100–3,200 and 1,000–2,300 m^2^/g for C_2_H_2_/CO_2_ and C_2_H_2_/CH_4_ separations, respectively. No obvious relation was found between selectivity, APS and porosity of MOFs as shown in Figures S4, S5 but lower porosities generally lead to higher selectivities. As a result, we concluded that pore sizes smaller than 10 Å, surface areas <2,000 m^2^/g and low porosities (0.41–0.64) provide a stronger confinement for the C_2_H_2_ molecules compared to CO_2_ and CH_4_ and lead to higher C_2_H_2_ selectivities. At that point, it is important to note that selectivity of a material is determined by the interplay of various factors and cannot be easily correlated to only a few structural properties as we attempted to do. For example, chemical composition and topology of MOFs strongly affect the affinity of materials for specific gas molecules but these correlations are very complex and they can be only captured if a very large number and variety of structures are investigated. The simple correlations that we demonstrated in Figures 5, 6 will provide useful information to accelerate the design of new high-performance MOFs for C_2_H_2_ separation applications.

**Figure 7:**
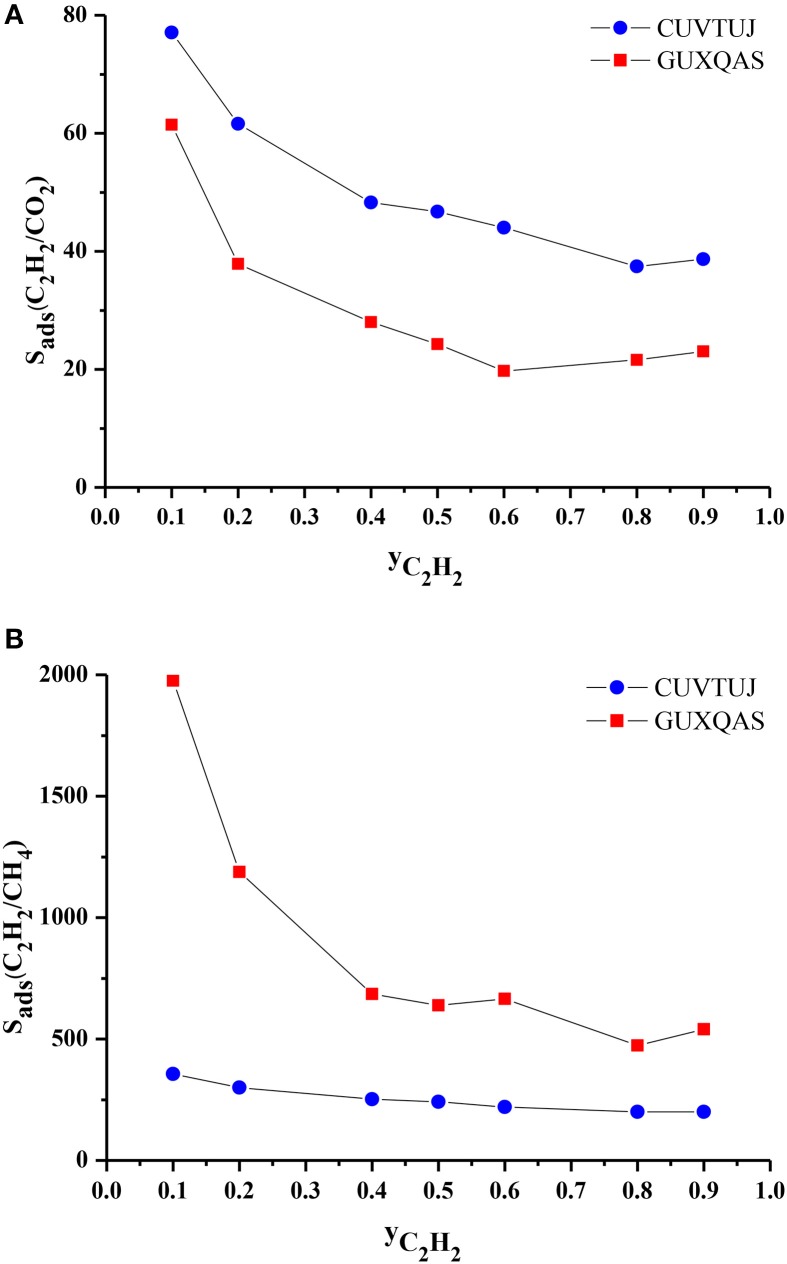
**(A)** C_2_H_2_/CO_2_ and **(B)** C_2_H_2_/CH_4_ selectivity of promising MOFs as a function of C_2_H_2_ composition in the bulk mixtures.

We finally investigated the effect of composition of the gas mixture on the separation performance of the MOFs. CUVTUJ and GUXQAS are the promising MOFs for C_2_H_2_/CO_2_ and C_2_H_2_/CH_4_ separations due to their high selectivities and high APSs. Outstanding performance of these two MOFs motivated us to investigate their separation performance for mixtures with different compositions. We performed GCMC simulation for these two MOFs at 1 bar, 298 K by changing the C_2_H_2_ mole fraction (y_C2H2_) in the bulk mixture. Figure 7A shows that as y_C2H2_ increases, selectivities of CUVTUJ and GUXQAS decrease. There are sharp decreases in the selectivities of both MOFs with increasing y_C2H2_ from 0.1 to 0.5. In this region, increasing y_C2H2_ results in an increase in the adsorbed C_2_H_2_ (x_C2H2_) and decrease in the adsorbed CO_2_ (x_CO2_). Since the selectivity was normalized by the bulk composition, C_2_H_2_/CO_2_ selectivities decrease with y_C2H2_. Figure 7B shows that GUXQAS has a very high C_2_H_2_/CH_4_ selectivity for mixtures with y_C2H2_ < 0.5. x_C2H2_ of GUXQAS does not change remarkably by increasing y_C2H2_, however x_CH4_ decreases resulting in sharp reductions in the C_2_H_2_/CH_4_ selectivities. The selectivity of CUVTUJ does not have a very significant dependence on the y_C2H2_ as can be seen from Figure 7B.

Finally, it is important to discuss the effect of force field selection on the results of molecular simulations. Generic force fields were found to be less successful in predicting gas adsorption in materials having strong binding sites, such as open metal sites (Getman et al., 2012). Specific force fields derived from quantum chemical calculations are required to describe the interactions between gas molecules and MOFs having open metal sites. The key challenge in developing such force fields using quantum chemistry calculations is selecting the appropriate level of theory and balancing it with the computational expense. There may be several MOFs having open metal sites and showing strong binding for C_2_H_2_ molecules but we did not define a specific force field for them and screened the MOFs using generic force fields. The value of our calculations is to efficiently identify the most promising MOF materials using generic, off-the-shelf-force fields, in a time-efficient manner. More detailed quantum chemistry calculations can be further performed for the best candidates to understand the underlying mechanism in future studies.

Our molecular simulations give no information about stability of MOFs, however an adsorbent should be stable in order to find place in practical applications. Therefore, we collected the stability information of the top three promising MOFs from their experimental synthesis articles. There is no specific stability information in the literature for CUVTUJ, OMORUE, and ONIXOZ. DOTSOV was reported to be thermally stable up to 240°C (Wu et al., 2008), UMUTIQ (Das et al., 2011) and GUXQAS (Ling et al., 2009) were reported to save their stabilities up to high temperatures. WAJHUM and WAJJAU were reported in the same experimental work and their thermal stabilities were investigated in detail (Xie et al., 2010). ODEZAB was reported to be thermally stable up to around 300°C after evacuating solvent molecules from its structure (Duan et al., 2016). Stabilities of the most promising materials identified in this work are most likely to be examined under practical gas separation experiments in future studies.

## Conclusions

Developing effective adsorbents for challenging separations of C_2_H_2_/CO_2_ and C_2_H_2_/CH_4_ is crucial in order to meet the requirement of high purity C_2_H_2_ in various industries. MOFs are strong candidates for storage of C_2_H_2_, but limited information was available about their C_2_H_2_ separation potentials. We performed the first large-scale molecular simulation study to examine the potential of 174 different MOF structures for separation of C_2_H_2_/CO_2_ and C_2_H_2_/CH_4_ mixtures. Several MOFs were identified to show high C_2_H_2_ selectivities. The top three most promising MOF adsorbents were computed to have C_2_H_2_/CO_2_ selectivities of 49, 47, 24, and C_2_H_2_/CH_4_ selectivities of 824, 684, 638 at 1 bar, 298 K. These are the highest C_2_H_2_ selectivities reported to date in the literature. Two MOFs, CUVTUJ and GUXQAS, were found to be very promising both for C_2_H_2_/CO_2_ and C_2_H_2_/CH_4_ separations leading to selectivities of 47 (242) and 24 (638), respectively. In addition to selectivity, other adsorbent evaluation metrics such as APS, S_sp_, R% were computed for all MOFs. Results showed that highly selective MOFs suffer from low R% and therefore choosing the best adsorbent material based on solely selectivity is not completely accurate. We also examined the structure-performance relations of MOFs and showed that MOFs with pore sizes <10 Å, surface areas <2,000 m^2^/g and low porosities (0.41–0.64) lead to higher C_2_H_2_ selectivities. We believe that these results will motivate extensive research on MOF adsorbents for C_2_H_2_ separation processes.

## Author contributions

AN: performed the molecular simulations and contributed to writing of the manuscript; SK: discussed the results and wrote the manuscript.

### Conflict of interest statement

The authors declare that the research was conducted in the absence of any commercial or financial relationships that could be construed as a potential conflict of interest.

## References

[B1] Alduhaish O. Li B. Arman H. Lin R.-B. Zhao J. C.-G. Chen B. (2017). A two-dimensional microporous metal–organic framework for highly selective adsorption of carbon dioxide and acetylene. Chin. Chem. Lett. 28, 1653–1658. 10.1016/j.cclet.2017.04.025

[B2] Altintas C. Keskin S. (2016). Computational screening of MOFs for C_2_H_6_/C_2_H_4_ and C_2_H_6_/CH_4_ separations. Chem. Eng. Sci. 139, 49–60. 10.1016/j.ces.2015.09.019

[B3] Bae Y. S. Snurr R. Q. (2011). Development and evaluation of porous materials for carbon dioxide separation and capture. Angew. Chem. 50, 11586–11596. 10.1002/anie.201101891 22021216

[B4] Ben-Mansour R. Habib M. A. Bamidele O. E. Basha M. Qasem N. A. A. Peedikakkal A. (2016). Carbon capture by physical adsorption: materials, experimental investigations and numerical modeling and simulations - a review. Appl. Energy 161, 225–255. 10.1016/j.apenergy.2015.10.011

[B5] Chen B. Siepmann J. I. (1999). Transferable potentials for phase equilibria. 3. explicit-hydrogen description of normal alkanes. J. Phys. Chem. B 103, 5370–5379. 10.1021/jp990822m

[B6] Chen D. M. Tian J. Y. Liu C. Sen C. M. Du M. (2016). Charge control in two isostructural anionic/cationic CoIICoordination frameworks for enhanced acetylene capture. Chem. Eur. J. 22, 15035–15041. 10.1002/chem.201603054 27593724

[B7] Chung Y. G. Camp J. Haranczyk M. Sikora B. J. Bury W. Krungleviciute V. (2014). Computation-ready, experimental metal-organic frameworks: a tool to enable high-throughput screening of nanoporous crystals. Chem. Mater. 26, 6185–6192. 10.1021/cm502594j

[B8] Chung Y. G. Gómez-Gualdrón D. A. Li P. Leperi K. T. Deria P. Zhang H.. (2016). *In silico* discovery of metal-organic frameworks for precombustion CO2 capture using a genetic algorithm. Sci. Adv. 2:e1600909. 10.1126/sciadv.1600909 27757420 PMC5065252

[B9] Colón Y. J. Snurr R. Q. (2014). High-throughput computational screening of metal–organic frameworks. Chem. Soc. Rev. 43, 5735–5749. 10.1039/C4CS00070F 24777001

[B10] Das M. C. Xu H. Xiang S. Zhang Z. Arman H. D. Qian G.. (2011). A new approach to construct a doubly interpenetrated microporous metal-organic framework of primitive cubic net for highly selective sorption of small hydrocarbon molecules. Chem. Eur. J. 17, 7817–7822. 10.1002/chem.201100350 21611990

[B11] Duan X. Cui Y. Yang Y. Qian G. (2016). A novel methoxy-decorated Metal-Organic Framework exhibiting high acetylene and carbon dioxide storage capacities. CrystEngComm 2, 1464–1469. 10.1039/C6CE02291J

[B12] Dubbeldam D. (2014). RASPA 2.0: Molecular software package for adsorption and diffusion in (Flexible) nanoporous materials (Basel). Mol. Simulat. 1–145.

[B13] Eddaoudi M. Kim J. Rosi N. Vodak D. Wachter J. Keeffe M. O. (2002). Systematic design of pore size and functionality in isoreticular MOFs and their application in methane storage published by : American association for the advancement of science linked references are available on JSTOR for this article : systematic design. Science 295, 469–472. 10.1126/science.1067208 11799235

[B14] Fischer M. Hoffmann F. Fröba M. (2010). New microporous materials for acetylene storage and C_2_H_2_/CO_2_ separation: insights from molecular simulations. Chemphyschem 11, 2220–2229. 10.1002/cphc.201000126 20540140

[B15] Foo M. L. Matsuda R. Hijikata Y. Krishna R. Sato H. Horike S.. (2016). An adsorbate discriminatory gate effect in a flexible porous coordination polymer for selective adsorption of CO_2_ over C_2_H_2_. J. Am. Chem. Soc. 138, 3022–3030. 10.1021/jacs.5b10491 26876504

[B16] Frenkel D. Smit B. (2002). Understanding Molecular Simulation: From Algorithms to Applications. San Diego, CA: Academic Press.

[B17] Getman R. B. Bae Y.-S. Wilmer C. E. Snurrr R. Q. (2012). Review and analysis of molecular simulations of methane, hydrogen, and acetylene storage in metal–organic frameworks. Chem. Rev. 112, 703–723. 10.1021/cr200217c 22188435

[B18] Groom C. R. Allen F. H. (2014). The cambridge structural database in retrospect and prospect. Angew. Chem. 53, 662–671. 10.1002/anie.201306438 24382699

[B19] Ji Y. Ding L. Cheng Y. Zhou H. Yang S. Li F. (2017). Understanding the effect of ligands on C_2_H_2_ storage and C_2_H_2_/CH_4_, C_2_H_2_/CO_2_ separation in metal-organic frameworks with open Cu(II) sites. J. Phys. Chem. C. 121, 24104–24113. 10.1021/acs.jpcc.7b08370

[B20] Keskin S. Liu J. Rankin R. B. Johnson J. K. Sholl D. S. (2009). Progress, opportunities, and challenges for applying atomically detailed modeling to molecular adsorption and transport in metal–organic framework materials. Ind. Eng. Chem. Res. 48, 2355–2371. 10.1021/ie800666s

[B21] Li H. Eddaoudi M. O'Keeffe M. Yaghi O. M. (1999). Design and synthesis of an exceptionally stable and highly porous metal-organic framework. Nature 402, 276–279.

[B22] Li J. R. Kupler R. J. Zhou H. C. (2009). Selective gas adsorption and separation in metal-organic frameworks. Chem. Soc. Rev. 38, 1477–1504. 10.1039/B802426J 19384449

[B23] Li J. R. Ma Y. McCarthy M. C. Sculley J. Yu J. Jeong H. K. (2011). Carbon dioxide capture-related gas adsorption and separation in metal-organic frameworks. Coord. Chem. Rev. 255, 1791–1823. 10.1016/j.ccr.2011.02.012

[B24] Li P. He Y. Zhao Y. Weng L. Wang H. Krishna R.. (2014). A Rod-packing microporous hydrogen-bonded organic framework for highly selective separation of C_2_H_2_/CO_2_ at room temperature. Angew. Chem. 54, 574–577. 10.1002/anie.201410077 25394888

[B25] Ling Y. Zhang L. Li J. Hu A. X. (2009). Three-fold-interpenetrated diamondoid coordination frameworks with torus links constructed by tetranuclear building blocks. Cryst. Growth Des. 9, 2043–2046. 10.1021/cg801188r

[B26] Mondloch J. E. Karagiaridi O. Farha O. K. Hupp J. T. (2013). Activation of metal- organic framework materials. CrystEngComm. 15, 9258–9264. 10.1039/c3ce41232f

[B27] Myers A. L. (2002). Thermodynamics of adsorption in porous materials. AIChE J. 48, 145–160. 10.1002/aic.690480115

[B28] Pang J. Jiang F. Wu M. Liu C. Su K. Lu W.. (2015). A porous metal-organic framework with ultrahigh acetylene uptake capacity under ambient conditions. Nat. Commun. 6:7575. 10.1038/ncomms8575 26123775 PMC4491824

[B29] Potoff J. J. Siepmann J. I. (2001). Vapor–liquid equilibria of mixtures containing alkanes, carbon dioxide, and nitrogen. AIChE J. 47, 1676–1682. 10.1002/aic.690470719

[B30] Rappé A. K. Casewit C. J. Colwell K. S. Goddard W. A. Skiff W. M. (1992). UFF, a full periodic table force field for molecular mechanics and molecular dynamics simulations. J. Am. Chem. Soc. 114, 10024–10035. 10.1021/ja00051a040

[B31] Rege S. Yang R. (2001). a Simple parameter for selecting an adsorbent for gas separation by pressure swing adsorption. Sep. Sci. Technol. 36, 3355–3365. 10.1081/SS-100107907

[B32] Sezginel K. B. Uzun A. Keskin S. (2015). Multivariable linear models of structural parameters to predict methane uptake in metal-organic frameworks. Chem. Eng. Sci. 124, 125–134. 10.1016/j.ces.2014.10.034

[B33] Sumer Z. Keskin S. (2016). Ranking of MOF Adsorbents for CO_2_ separations: a molecular simulation study. Ind. Eng. Chem. Res. 55, 10404–10419. 10.1021/acs.iecr.6b02585

[B34] van Gunsteren W. Daura X. Hansen N. Mark A. Oostenbrink C. Riniker S.. (2017). Validation of molecular simulation: an overview of issues. Angew. Chem. 57, 884–902. 10.1002/anie.201702945 28682472

[B35] Wen H.-M. Wang H. Li B. Cui Y. Wang H. Qian G.. (2016). A microporous metal–organic framework with lewis basic nitrogen sites for high C_2_H_2_ storage and significantly enhanced C_2_H_2_/CO_2_ separation at ambient conditions. Inorg. Chem. 55, 7214–7218. 10.1021/acs.inorgchem.6b00748 27176900

[B36] Willems T. F. Rycroft C. H. Kazi M. Meza J. C. Haranczyk M. (2012). Algorithms and tools for high-throughput geometry-based analysis of crystalline porous materials. Microporous Mesoporous Mater. 149, 134–141. 10.1016/j.micromeso.2011.08.020

[B37] Wilmer C. E. Kim K. C. Snurr R. Q. (2012). An extended charge equilibration method. J. Phys. Chem. Lett. 3, 2506–2511. 10.1021/jz3008485 26292141

[B38] Wu Y. Kobayashi A. Halder G. J. Peterson V. K. Chapman K. W. Lock N. (2008). Negative thermal expansion in the metal-organic framework material Cu 3(1,3,5-benzenetricarboxylate)2. Angew. Chem. 47, 8929–8932. 10.1002/anie.200803925 18850600

[B39] Xiang S. C. Zhou W. Gallegos J. M. Liu Y. Chen B. L. (2009). Exceptionally high acetylene uptake in a microporous metal-organic framework with open metal sites. J. Am. Chem. Soc. 131, 12415–12419. 10.1021/ja904782h 19705919

[B40] Xiang S. Zhou W. Zhang Z. Green M. A. Liu Y. Chen B. (2010). Open metal sites within isostructural metal-organic frameworks for differential recognition of acetylene and extraordinarily high acetylene storage capacity at room temperature. Angew. Chem. Int. Edn. 49, 4615–4618. 10.1002/anie.201000094 20491101

[B41] Xie L. H. Lin J. Bin L. X. M. Wang Y. Zhang W. X. Zhang J. P.. (2010). Porous coordination polymer with flexibility imparted by coordinatively changeable lithium ions on the pore surface. Inorg. Chem. 49, 1158–1165. 10.1021/ic902077j 20050697

[B42] Xu H. He Y. Zhang Z. Xiang S. Cai J. Cui Y. (2013). A microporous metal–organic framework with both open metal and Lewis basic pyridyl sites for highly selective C_2_H_2_/CH_4_ and C_2_H_2_/CO _2_ gas separation at room temperature. J. Mater. Chem. A 1, 77–81. 10.1039/C2TA00155A

[B43] Yeganegi S. Gholami M. Sokhanvaran V. (2017). Molecular simulations of adsorption and separation of acetylene and methane and their binary mixture on MOF-5, HKUST-1 and MOF-505 metal–organic frameworks. Mol. Simul. 43, 260–266. 10.1080/08927022.2016.1262036

[B44] Zhang C. Lan Y. Guo X. Yang Q. Zhong C. (2017). Materials genomics-guided ab initio screening of MOFs with open copper sites for acetylene storage. AIChE J. [Epub ahead of print]. 10.1002/aic.16025

[B45] Zhang Z. Xiang S. Chen B. (2011). Microporous metal–organic frameworks for acetylene storage and separation. CrystEngComm 13, 5983–5992. 10.1039/c1ce05437f

