# Supplementary material for: Computational Screening of MOFs for Acetylene Separation
Source: Front Chem. 2018 Feb 27;6:36. doi: 10.3389/fchem.2018.00036 (PMC5835272; doi:10.3389/fchem.2018.00036)
Supplement: Supplementary file 1 [file DataSheet1.DOCX]

Supplementary Material

Computational Screening of MOFs for Acetylene Separation

Ayda Nemati Vesali Azar *, Seda Keskin

*** Correspondence:** Seda Keskin: [skeskin@ku.edu.tr](mailto:skeskin@ku.edu.tr)

**Table S1.** Structural properties of MOFs

| **No.** | **CCDC refcode** | **Metal sites** | **LCD** (Å) | **PLD** (Å) | **Surface Area** (m^2^/g) | **Pore volume** (cm^3^/g) | **Porosity** | **Density** (g/cm^3^) |
| --- | --- | --- | --- | --- | --- | --- | --- | --- |
| 1 | ACENIF | Zn | 5.50 | 4.18 | 726.28 | 0.41 | 0.54 | 1.31 |
| 2 | AFUJUI | Mn | 6.22 | 3.91 | 833.45 | 0.45 | 0.52 | 1.15 |
| 3 | ALICEE | Cu | 8.89 | 8.88 | 662.93 | 0.34 | 0.58 | 1.70 |
| 4 | ALICII | Cu | 9.46 | 9.45 | 798.66 | 0.41 | 0.62 | 1.51 |
| 5 | ALOLOD | In | 7.87 | 5.03 | 1749.37 | 0.67 | 0.62 | 0.92 |
| 6 | BEDHOJ | Ni | 4.76 | 4.42 | 445.99 | 0.36 | 0.48 | 1.31 |
| 7 | BENXOJ | Zn | 4.10 | 3.48 | 705.97 | 0.34 | 0.50 | 1.45 |
| 8 | BIYTEJ | Zn | 6.55 | 6.39 | 626.66 | 0.39 | 0.54 | 1.39 |
| 9 | BONMAT | Ni | 6.20 | 5.03 | 1989.30 | 0.75 | 0.64 | 0.86 |
| 10 | BOWSIQ | Cu | 5.03 | 3.25 | 782.59 | 0.40 | 0.48 | 1.21 |
| 11 | BUSMUY | Co | 7.34 | 5.90 | 558.00 | 0.35 | 0.50 | 1.43 |
| 12 | BUSNAF | Co | 7.08 | 5.44 | 221.13 | 0.16 | 0.45 | 2.88 |
| 13 | BUVWOF02 | Fe | 7.03 | 5.39 | 305.90 | 0.20 | 0.47 | 2.31 |
| 14 | BUVXOG01 | Fe | 7.08 | 5.43 | 471.56 | 0.32 | 0.47 | 1.50 |
| 15 | BUVYIB01 | Fe | 10.82 | 8.63 | 1876.83 | 0.65 | 0.71 | 1.09 |
| 16 | CAGSAG | Cu | 8.86 | 4.53 | 1144.42 | 0.55 | 0.59 | 1.08 |
| 17 | CAYDOX | Cu | 5.15 | 4.99 | 365.47 | 0.31 | 0.47 | 1.49 |
| 18 | CAYGIU | Cu | 5.16 | 4.99 | 366.05 | 0.31 | 0.47 | 1.49 |
| 19 | CELZUQ | Al | 6.04 | 3.76 | 734.78 | 0.48 | 0.54 | 1.12 |
| 20 | CUGVUW | Cu | 6.47 | 4.58 | 233.51 | 0.19 | 0.45 | 2.38 |
| 21 | CUVTUJ | Co | 11.50 | 10.88 | 1226.33 | 0.54 | 0.64 | 1.18 |
| 22 | DAWMUL | Cu | 28.67 | 14.33 | 5751.60 | 3.34 | 0.89 | 0.27 |
| 23 | DEJROB | Co | 5.53 | 3.56 | 1072.41 | 0.45 | 0.51 | 1.14 |
| 24 | DIDBID | Co | 9.15 | 5.88 | 1472.39 | 0.64 | 0.59 | 0.93 |
| 25 | DIDBOJ | Co | 9.16 | 5.75 | 1460.53 | 0.65 | 0.60 | 0.92 |
| 26 | DONNAW1 | Ga | 6.67 | 6.40 | 1202.68 | 0.51 | 0.60 | 1.16 |
| 27 | DORDUK | In | 4.54 | 4.13 | 335.64 | 0.35 | 0.49 | 1.43 |
| 28 | DOTSOV | Cu | 13.29 | 6.69 | 2507.25 | 0.72 | 0.63 | 0.88 |
| 29 | EBAMOL | Ni | 7.96 | 4.27 | 878.96 | 0.57 | 0.60 | 1.05 |
| 30 | EBUQEX | Na | 5.33 | 3.59 | 1357.39 | 0.50 | 0.51 | 1.03 |
| 31 | ECAHIB | Cu | 6.60 | 4.45 | 402.86 | 0.28 | 0.47 | 1.66 |
| 32 | EHUFAP | Cu | 4.35 | 3.32 | 99.02 | 0.28 | 0.42 | 1.52 |
| 33 | EJOGOZ | Cu | 8.56 | 7.68 | 896.98 | 0.45 | 0.55 | 1.23 |
| 34 | EXOTUH | Cd | 6.06 | 4.41 | 1280.36 | 0.51 | 0.62 | 1.23 |
| 35 | EYOQEP | Cd | 6.74 | 4.17 | 896.18 | 0.48 | 0.55 | 1.15 |
| 36 | FAGREM | Zn | 6.99 | 5.61 | 736.30 | 0.35 | 0.53 | 1.53 |
| 37 | FALTUK | Cu | 14.44 | 6.65 | 3281.48 | 1.13 | 0.75 | 0.67 |
| 38 | FANWIC | Zn | 9.50 | 8.17 | 1041.99 | 0.53 | 0.58 | 1.10 |
| 39 | FANWOI | Zn | 9.82 | 8.59 | 1051.54 | 0.54 | 0.58 | 1.09 |
| 40 | FAYRAA | Zn | 11.92 | 6.64 | 2486.73 | 1.00 | 0.72 | 0.72 |
| 41 | FEBQIO | Mn | 9.30 | 6.68 | 2060.95 | 0.75 | 0.67 | 0.89 |
| 42 | FEHCOM | Zn | 7.42 | 6.75 | 1507.87 | 0.66 | 0.63 | 0.95 |
| 43 | FIFMUE | Co | 4.62 | 3.81 | 196.99 | 0.37 | 0.48 | 1.31 |
| 44 | GEDSIT | Nd | 4.61 | 2.75 | 1008.21 | 0.32 | 0.46 | 1.46 |
| 45 | GIQHAQ | Cd | 14.51 | 14.41 | 966.97 | 0.53 | 0.63 | 1.19 |
| 46 | GITTIN | Co | 11.24 | 3.73 | 1187.35 | 0.55 | 0.61 | 1.09 |
| 47 | GITVIP01 | Zn | 17.01 | 5.47 | 1009.11 | 0.55 | 0.63 | 1.15 |
| 48 | GUPBEZ | Cu | 7.29 | 4.38 | 143.00 | 0.17 | 0.44 | 2.54 |
| 49 | GUPCUQ01 | Cd | 12.59 | 3.83 | 1242.31 | 0.57 | 0.65 | 1.14 |
| 50 | GUPDIF | Cd | 4.75 | 3.85 | 344.81 | 0.45 | 0.54 | 1.20 |
| 51 | GUSPUG | Cu | 13.36 | 11.38 | 586.79 | 0.38 | 0.55 | 1.45 |
| 52 | GUXPUL | Zn | 7.58 | 6.37 | 688.00 | 0.24 | 0.38 | 1.60 |
| 53 | GUXQAS | Cu | 8.85 | 6.07 | 632.24 | 0.22 | 0.48 | 2.18 |
| 54 | HANWAW | Cu | 7.53 | 4.96 | 2034.92 | 0.76 | 0.67 | 0.88 |
| 55 | HEBZAR | Br,Zn | 7.78 | 3.22 | 737.54 | 0.31 | 0.53 | 1.72 |
| 56 | HEBZEV | Zn | 7.74 | 3.68 | 637.19 | 0.37 | 0.54 | 1.47 |
| 57 | HIHJUF | Zn | 5.85 | 4.89 | 420.68 | 0.34 | 0.50 | 1.48 |
| 58 | HIYGIH | Cu | 17.09 | 5.22 | 2221.12 | 0.95 | 0.73 | 0.77 |
| 59 | HOHMIB | Zn | 13.19 | 11.42 | 3880.50 | 1.44 | 0.78 | 0.54 |
| 60 | HUTZAX01 | Zn | 5.15 | 3.92 | 224.74 | 0.32 | 0.46 | 1.42 |
| 61 | IMIDZB02 | Zn | 4.36 | 2.43 | 236.44 | 0.29 | 0.45 | 1.54 |
| 62 | ISOJOQ | In | 5.96 | 4.18 | 1665.14 | 0.62 | 0.61 | 0.99 |
| 63 | KIYMIQ | Na | 5.47 | 5.36 | 202.89 | 0.25 | 0.34 | 1.33 |
| 64 | KOJCUI | Mn | 5.42 | 4.82 | 288.07 | 0.26 | 0.40 | 1.52 |
| 65 | LAJKUE | Cs | 16.15 | 6.68 | 959.67 | 0.54 | 0.58 | 1.08 |
| 66 | LAJLEP | Rb | 16.84 | 7.14 | 1196.30 | 0.59 | 0.59 | 1.00 |
| 67 | LASPOM | Cu | 4.65 | 3.45 | 449.22 | 0.31 | 0.42 | 1.37 |
| 68 | LASYOU | Cu | 10.06 | 3.58 | 1928.14 | 0.61 | 0.66 | 1.07 |
| 69 | LAYQUA | Cu | 7.96 | 5.76 | 2805.47 | 0.91 | 0.71 | 0.77 |
| 70 | LAYRAH | Cu | 7.73 | 4.71 | 1786.16 | 0.58 | 0.67 | 1.15 |
| 71 | LAZPOU | Cu | 7.41 | 4.11 | 913.64 | 0.42 | 0.59 | 1.39 |
| 72 | LAZSIQ | Cu | 5.42 | 2.94 | 1615.63 | 0.44 | 0.54 | 1.23 |
| 73 | LEBCUT | Cu | 7.49 | 4.13 | 1186.22 | 0.45 | 0.63 | 1.40 |
| 74 | LELMEW | Cu | 13.36 | 4.43 | 1133.94 | 0.54 | 0.61 | 1.14 |
| 75 | LETFEW | Cu | 5.62 | 4.46 | 325.69 | 0.22 | 0.47 | 2.12 |
| 76 | LUXDEO | Ag | 5.13 | 2.58 | 1701.24 | 0.44 | 0.52 | 1.19 |
| 77 | MIBQAR | Zn | 15.10 | 7.96 | 3711.23 | 1.38 | 0.81 | 0.59 |
| 78 | MIHHIU | Cd | 5.23 | 3.48 | 468.18 | 0.34 | 0.45 | 1.30 |
| 79 | MIYGOR | Ag | 6.66 | 4.13 | 552.35 | 0.33 | 0.54 | 1.61 |
| 80 | MIYGUX | Cu | 6.46 | 3.90 | 512.87 | 0.40 | 0.54 | 1.36 |
| 81 | MOXJOZ | Cu | 10.21 | 2.89 | 1906.83 | 0.60 | 0.58 | 0.97 |
| 82 | MUCLOM | Li | 10.23 | 2.86 | 2216.32 | 0.69 | 0.57 | 0.83 |
| 83 | NEXXIZ | Li | 9.74 | 9.22 | 865.33 | 0.62 | 0.55 | 0.89 |
| 84 | NEYYOG | Cu | 12.40 | 10.67 | 260.50 | 0.22 | 0.50 | 2.30 |
| 85 | NICJOA | Cu,Ba | 5.24 | 4.20 | 103.50 | 0.14 | 0.35 | 2.48 |
| 86 | NUTQAV | Cu | 10.91 | 4.12 | 2675.74 | 0.89 | 0.72 | 0.82 |
| 87 | NUTQEZ | Cu | 11.69 | 4.31 | 1245.06 | 0.41 | 0.71 | 1.72 |
| 88 | ODEZAB | Cu | 7.17 | 4.44 | 1076.89 | 0.37 | 0.68 | 1.81 |
| 89 | OFERUN | Zn | 11.51 | 3.42 | 1727.64 | 0.65 | 0.63 | 0.96 |
| 90 | OFIWIK01 | Zn | 5.20 | 2.86 | 173.51 | 0.25 | 0.42 | 1.71 |
| 91 | OLOYOF | Cu | 10.67 | 3.83 | 2341.18 | 0.84 | 0.71 | 0.85 |
| 92 | OMORUE | Cu | 6.49 | 5.35 | 2101.39 | 0.73 | 0.67 | 0.92 |
| 93 | ONIXOZ | Cu | 9.94 | 9.19 | 1809.28 | 0.67 | 0.67 | 1.00 |
| 94 | OSIWOD01 | Ag | 4.60 | 4.04 | 130.96 | 0.22 | 0.44 | 1.98 |
| 95 | OSOTIB | Cu | 11.04 | 4.63 | 2560.84 | 0.77 | 0.71 | 0.93 |
| 96 | OTARIL | Zn | 5.67 | 3.91 | 556.50 | 0.42 | 0.49 | 1.17 |
| 97 | OYODAI | Zn | 9.58 | 2.48 | 1840.92 | 0.62 | 0.59 | 0.95 |
| 98 | OYODEM | Zn | 8.97 | 5.23 | 956.97 | 0.54 | 0.56 | 1.02 |
| 99 | PAVLUU | Ag | 4.64 | 4.30 | 459.72 | 0.41 | 0.55 | 1.35 |
| 100 | PEGBEK | Zn | 4.25 | 3.44 | 563.33 | 0.33 | 0.48 | 1.43 |
| 101 | PEHBIO | Rh | 5.33 | 3.83 | 880.75 | 0.55 | 0.63 | 1.14 |
| 102 | PEJNOJ | Zn | 6.26 | 5.56 | 2986.99 | 0.89 | 0.71 | 0.80 |
| 103 | PURQUP | Cu | 10.70 | 8.13 | 2027.62 | 0.79 | 0.69 | 0.87 |
| 104 | QABVEW | Co,Cu | 7.68 | 3.03 | 2003.93 | 0.61 | 0.62 | 1.02 |
| 105 | QEDZEG | Zn | 4.67 | 4.35 | 285.13 | 0.39 | 0.50 | 1.27 |
| 106 | RAXCAW | Zn | 5.03 | 3.12 | 486.20 | 0.34 | 0.48 | 1.38 |
| 107 | RAYMAH | Zn | 6.61 | 4.78 | 849.77 | 0.43 | 0.53 | 1.22 |
| 108 | REGXOS | Co | 19.51 | 7.12 | 1538.26 | 0.72 | 0.66 | 0.92 |
| 109 | REGYOT | Cu | 17.69 | 10.17 | 1699.08 | 1.07 | 0.74 | 0.70 |
| 110 | REPCIZ | Cd | 8.97 | 8.08 | 1845.37 | 0.69 | 0.68 | 0.98 |
| 111 | REWNEO | Cu | 19.42 | 5.07 | 3388.78 | 1.26 | 0.77 | 0.61 |
| 112 | REYNUG | Zn | 3.11 | 2.85 | 130.23 | 0.18 | 0.35 | 1.88 |
| 113 | ROGMEG | Zn | 12.37 | 9.88 | 4271.79 | 1.45 | 0.78 | 0.54 |
| 114 | SAHYIK | Zn | 14.95 | 7.84 | 3606.48 | 1.33 | 0.81 | 0.60 |
| 115 | SAHYOQ | Zn | 15.10 | 7.97 | 3690.90 | 1.38 | 0.81 | 0.59 |
| 116 | SAPJEA | Zn,Ga | 6.49 | 6.32 | 219.92 | 0.08 | 0.38 | 5.05 |
| 117 | SAPJIE | Zn,Al | 6.66 | 6.48 | 228.20 | 0.08 | 0.34 | 4.38 |
| 118 | SARBOE | Zn | 5.05 | 3.84 | 616.44 | 0.45 | 0.54 | 1.20 |
| 119 | SEGBIR | Cu | 10.06 | 9.57 | 339.42 | 0.23 | 0.52 | 2.26 |
| 120 | SEGBOX | Cu | 10.03 | 9.54 | 479.92 | 0.32 | 0.52 | 1.63 |
| 121 | SUTBIT | Ni | 8.39 | 7.96 | 1862.36 | 0.77 | 0.66 | 0.86 |
| 122 | TEPGUS | Zn | 4.97 | 3.88 | 482.85 | 0.39 | 0.51 | 1.29 |
| 123 | TEQTAM | Cd | 6.97 | 5.82 | 832.78 | 0.41 | 0.52 | 1.26 |
| 124 | TEWFUY01 | Cu | 5.19 | 3.83 | 461.33 | 0.41 | 0.49 | 1.20 |
| 125 | TIGDOD | Zn | 6.09 | 4.62 | 1486.64 | 0.64 | 0.60 | 0.94 |
| 126 | TUDHUW | Zn | 11.39 | 3.40 | 1766.48 | 0.69 | 0.64 | 0.93 |
| 127 | TUDJAE | Zn | 11.46 | 3.50 | 1824.81 | 0.71 | 0.65 | 0.91 |
| 128 | TUDJEI | Zn | 11.46 | 3.50 | 1824.81 | 0.71 | 0.64 | 0.91 |
| 129 | TUDJIM | Zn | 11.40 | 3.42 | 1776.40 | 0.69 | 0.64 | 0.93 |
| 130 | TUDJOS | Zn | 11.49 | 3.95 | 1450.52 | 0.71 | 0.65 | 0.91 |
| 131 | TUDJUY | Zn | 11.45 | 3.49 | 1814.82 | 0.70 | 0.64 | 0.92 |
| 132 | TUDKAF | Zn | 11.49 | 3.52 | 1832.82 | 0.71 | 0.65 | 0.91 |
| 133 | TUDKEJ | Zn | 11.41 | 3.43 | 1775.80 | 0.69 | 0.64 | 0.92 |
| 134 | TUDMAH | Ni | 6.63 | 4.44 | 2346.82 | 0.80 | 0.65 | 0.81 |
| 135 | TUDMEL | Ni | 5.74 | 2.59 | 1585.95 | 0.49 | 0.53 | 1.07 |
| 136 | TUSGUJ | Zn,Ag | 11.79 | 4.89 | 1755.08 | 0.74 | 0.65 | 0.88 |
| 137 | UFANEV | Ag | 6.19 | 5.42 | 972.98 | 0.41 | 0.55 | 1.35 |
| 138 | UMUXAC | Cu | 6.23 | 4.07 | 102.89 | 0.16 | 0.43 | 2.75 |
| 139 | UNIGEE | Zn | 14.93 | 7.84 | 3598.88 | 1.33 | 0.81 | 0.61 |
| 140 | UWAGAB03 | Zn | 11.63 | 8.54 | 3286.29 | 1.32 | 0.76 | 0.58 |
| 141 | UWUTIQ | Zn | 4.08 | 3.10 | 806.00 | 0.31 | 0.41 | 1.33 |
| 142 | UXEHIP | Zn | 6.09 | 4.82 | 1371.14 | 0.62 | 0.58 | 0.94 |
| 143 | UYAQAN | Mg | 7.19 | 5.37 | 742.47 | 0.40 | 0.46 | 1.14 |
| 144 | UYAQER | Zn | 7.14 | 5.65 | 428.67 | 0.30 | 0.46 | 1.53 |
| 145 | VAGMIB | Zn | 11.07 | 10.14 | 4767.71 | 1.76 | 0.81 | 0.46 |
| 146 | VAGMOH | Zn | 9.56 | 7.67 | 4682.62 | 1.60 | 0.79 | 0.49 |
| 147 | VEHKEA | Zn | 10.10 | 8.73 | 1693.12 | 0.66 | 0.71 | 1.07 |
| 148 | VETSUK | Co | 20.38 | 12.04 | 1859.02 | 1.20 | 0.75 | 0.62 |
| 149 | VEVJUD | Cu | 11.96 | 9.22 | 2230.79 | 0.82 | 0.76 | 0.94 |
| 150 | VIZRIH | Zn | 5.69 | 4.30 | 230.43 | 0.27 | 0.42 | 1.59 |
| 151 | VOGTIV | Mg | 11.71 | 10.91 | 1757.95 | 0.71 | 0.65 | 0.92 |
| 152 | VURMOL | Zn | 13.72 | 6.45 | 3190.70 | 1.14 | 0.78 | 0.69 |
| 153 | WAFKAQ | Zn | 8.03 | 5.52 | 2136.97 | 0.73 | 0.66 | 0.91 |
| 154 | WAFKIY | Zn | 8.63 | 7.06 | 2077.24 | 0.75 | 0.67 | 0.89 |
| 155 | WAJHOG | Zn,Li | 5.47 | 4.31 | 549.97 | 0.39 | 0.52 | 1.32 |
| 156 | WAJHUM | Zn,Li | 7.48 | 7.27 | 1043.28 | 0.45 | 0.56 | 1.23 |
| 157 | WAJJAU | Zn,Li | 7.48 | 7.27 | 1018.43 | 0.45 | 0.56 | 1.23 |
| 158 | WAJJEY | Zn,Li | 7.48 | 7.27 | 1054.78 | 0.45 | 0.56 | 1.23 |
| 159 | WAJJOI | Zn,Li | 7.49 | 7.26 | 1047.67 | 0.45 | 0.56 | 1.23 |
| 160 | WALCIX | Zn | 4.18 | 2.99 | 947.80 | 0.28 | 0.48 | 1.71 |
| 161 | WEMFUR | Be | 5.36 | 3.39 | 620.23 | 0.34 | 0.43 | 1.26 |
| 162 | WEMGAY | Be | 5.51 | 4.35 | 1012.62 | 0.49 | 0.54 | 1.10 |
| 163 | WEMXIX | Cu | 6.24 | 5.68 | 551.68 | 0.29 | 0.44 | 1.51 |
| 164 | WETPES | Cu | 10.55 | 5.57 | 2399.46 | 0.93 | 0.72 | 0.77 |
| 165 | XAGCEO | Co | 5.65 | 2.54 | 904.90 | 0.24 | 0.43 | 1.81 |
| 166 | XALROT | In | 5.95 | 4.26 | 1459.88 | 0.65 | 0.63 | 0.97 |
| 167 | XENZUN | V,Na | 9.38 | 3.79 | 857.55 | 0.52 | 0.57 | 1.09 |
| 168 | XIJNUA | Cd | 16.10 | 15.60 | 1416.20 | 0.79 | 0.82 | 1.04 |
| 169 | XIRWEB | Cs,Cu | 7.22 | 4.29 | 416.69 | 0.35 | 0.49 | 1.42 |
| 170 | XUNJEW | Zn | 7.71 | 4.19 | 794.81 | 0.40 | 0.54 | 1.35 |
| 171 | YARFII | Zn | 5.61 | 3.63 | 1516.15 | 0.53 | 0.55 | 1.04 |
| 172 | YEYVII | Ag | 6.31 | 3.75 | 460.56 | 0.34 | 0.54 | 1.59 |
| 173 | YIWLOF | Cd | 6.84 | 5.42 | 1363.15 | 0.53 | 0.69 | 1.30 |
| 174 | YOZBUL01 | Zn | 12.19 | 10.31 | 1208.93 | 0.58 | 0.64 | 1.10 |
| 175 | YUKBIP | Al | 7.46 | 5.14 | 327.97 | 0.22 | 0.46 | 2.09 |
| 176 | ZARMAI | Zn | 10.70 | 2.88 | 1919.07 | 0.64 | 0.64 | 1.00 |
| 177 | ZESTIC | Cd | 5.12 | 3.90 | 626.74 | 0.48 | 0.53 | 1.10 |
| 178 | ZIGFEC | Cr | 12.83 | 6.85 | 2418.78 | 0.90 | 0.72 | 0.80 |

**Table S2.** Potential parameters and partial charges of C_2_H_2_, CO_2_ and CH_4_ molecules.

| Gas models | Atoms | σ (Å) | ε (K) | Charge | Bond lengths, d (Å) |
| --- | --- | --- | --- | --- | --- |
| Acetylene 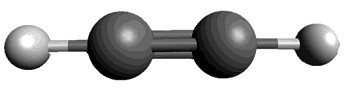 d **_C─C_**  d **_C─H_**  d **_C─H_**  **-q**  **-q**  **+q**  **+q** | C–C_2_H_2_ | 3.8 | 57.875 | -0.278 | 1.2111 (C−C) |
|  | H–C_2_H_2_ | − | − | +0.278 | 1.0712 (C−H) |
| Carbon dioxide 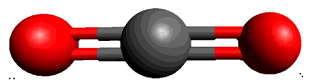 d **_C─O_**  d **_C─O_**  **-q**  **-q**  **+2q** | C−CO_2_ | 2.8 | 27.0 | +0.70 | 1.1625 (C−O) |
|  | O−CO_2_ | 3.05 | 79.0 | -0.35 | - |
| 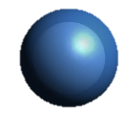Methane | United atom | 3.73 | 148 | Non-polar | - |
|  |  |  |  |  |  |

**Table S3.** Standard deviations of gas adsorption data obtained from GCMC simulations at 1 bar.

| C_2_H_2_/CH_4_ | | | |
| --- | --- | --- | --- |
| MOFs | C_2_H_2_  (molecules/unit cell) | deviation  (molecules/unit cell) | %error |
| CUVTUJ | 22.28 | 0.18 | 0.81 |
| ODEZAB | 76.91 | 0.28 | 0.36 |
| ONIXOZ | 28.34 | 0.28 | 0.99 |
| C_2_H_2_/CO_2_ | | | |
| MOFs | C_2_H_2_  (molecules/unit cell) | deviation  (molecules/unit cell) | %error |
| CUVTUJ | 22.01 | 0.58 | 2.64 |
| OMORUE | 25.18 | 0.77 | 3.06 |
| DOTSOV | 52.36 | 0.95 | 1.81 |

**Figure S1.** Number of adsorbed C_2_H_2_ molecules vs. number of equilibration cycles for (a)C_2_H_2_/CO_2_ and (b)C_2_H_2_/CH_4_ mixtures.

**Figure S2.** Comparison of experimentally measured and simulated C_2_H_2_ adsorption isotherm data for IRMOF-1(circles) and CuBTC (squares). Filled symbols are the experimental data taken from Xiang et al., 2009, half-filled symbols are taken from a simulation study of Fischer et al., 2010 and open symbols are the results of this work.

**Figure S3.** APS and surface areas of MOFs for (a) C_2_H_2_/CO_2_ and (b) C_2_H_2_/CH_4_ mixtures.

Figure S4. S_ads_ and porosity of MOFs for (a) C_2_H_2_/CO_2_ and (b) C_2_H_2_/CH_4_ mixtures.

Figure S5. APS and porosity of MOFs for (a) C_2_H_2_/CO_2_ and (b) C_2_H_2_/CH_4_ mixtures.

Fischer, M., Hoffmann, F., and Fröba, M. (2010). New microporous materials for acetylene storage and C_2_H_2_/CO_2_ separation: Insights from molecular simulations. *ChemPhysChem* 11, 2220-2229. doi:10.1002/cphc.201000126.

Xiang, S. C., Zhou, W., Gallegos, J. M., Liu, Y., and Chen, B. L. (2009). Exceptionally High Acetylene Uptake in a Microporous Metal-Organic Framework with Open Metal Sites. *J. Am. Chem. Soc.* 131, 12415-12419. doi:10.1021/ja904782h.
